# Falls Do Not Just Break Bones--They Break Lives: World Spinal Cord Injury Day (September 5, 2025) and Indian Perspectives

**DOI:** 10.7759/cureus.110329

**Published:** 2026-06-05

**Authors:** Raktim Swarnakar

**Affiliations:** 1 Physical Medicine and Rehabilitation, National Cancer Institute (NCI-AIIMS) Jhajjar Campus, All India Institute of Medical Sciences, New Delhi, IND

**Keywords:** disability, fall prevention, public-health, rehabilitation, spinal cord injury

## Abstract

Spinal cord injury (SCI) is a catastrophic condition that often results in lifelong disability, loss of independence, and profound social and economic consequences. While road traffic accidents remain a major cause, falls are increasingly emerging as a significant and preventable contributor to SCI, particularly in India. The theme of World SCI Day 2025--“Fall Prevention, Spinal Cord Protection”--highlights the urgent need to address this issue through proactive measures. Effective fall prevention requires a multipronged approach that combines home and community safety interventions, medical screening, occupational safeguards, and the integration of technology such as wearable devices and mobile applications. Equally important is the role of health systems in incorporating fall risk screening into routine care and ensuring access to rehabilitation and preventive services. For India, prevention represents not only a clinical necessity but also a social and economic imperative, as a single fall-related SCI can alter the trajectory of entire families. This editorial emphasizes that falls do not merely break bones--they break lives. Strengthening fall prevention strategies and embedding them into public health and rehabilitation frameworks can preserve dignity, mobility, and quality of life while reducing the broader societal burden of SCI.

## Editorial

The adage “prevention is better than cure” holds especially true for spinal cord injury (SCI), one of the most devastating medical conditions, leading to lifelong disability, reduced quality of life, and a significant socioeconomic burden. Each year, on September 5, World Spinal Cord Injury (SCI) Day draws global attention to this condition, with themes that emphasize both prevention and care. The 2025 theme--“Fall Prevention, Spinal Cord Protection”--is particularly relevant to India, where falls are increasingly recognized as a leading, yet preventable, cause of SCI [1,2].

Globally, falls account for 20-30% of SCI cases, but in India, the proportion is considerably higher in certain subgroups, especially among older adults and rural populations [2,3]. Risk factors in India are often compounded by osteoporosis, poor vision, polypharmacy, malnutrition, unsafe housing structures, and lack of awareness [4]. In rural areas, occupational practices such as climbing trees, carrying heavy loads, or working on unstable rooftops further increase the likelihood of high-energy falls, leading to catastrophic injuries [4]. Among older adults, even a seemingly minor bathroom slip may result in permanent paralysis [4].

The tragedy of fall-related SCI lies in its preventability. Evidence supports a range of low-cost, practical interventions: home safety modifications (grab bars, slip-resistant flooring, and proper lighting); medical measures (osteoporosis screening and treatment, regular vision checks, and medication review); community-based approaches (strength and balance training, physiotherapy, and awareness campaigns); and occupational safety initiatives (protective equipment and safer practices in agriculture and construction) [5]. Fall-prevention programs must also address the needs of people with disabilities, emphasizing safe and accessible environments.

In the Indian context, prevention requires culturally and economically appropriate strategies. Rural households should adopt safer construction practices, such as installing stair railings and secure ladders, while avoiding unsafe practices like unprotected tree climbing [1,5]. Urban homes should integrate non-slippery bathroom tiles, improved corridor and staircase lighting, and elderly friendly designs [5]. Community health workers can play a crucial role by screening older adults for fall risk and educating families about simple preventive measures [5]. Schools and workplaces can contribute through safety education, while local governments must ensure safe pavements, public toilets, and transport systems [5]. Technology, including mobile apps, wearable devices, and assistive tools, offers additional opportunities for fall prevention [1]. Such context-specific, low-cost interventions could significantly reduce fall-related SCI in India.

Older adults tend to be more aware of environmental and behavioral risks but often lack knowledge about biological and socioeconomic factors [4]. Enhancing awareness in these areas can help them better recognize their vulnerabilities and take preventive action [4]. In addition, simple clinical assessments can be used to estimate fall risk, while individualized multidomain interventions are effective for high-risk community-dwelling older adults [5]. In hospitals and care homes, where all older individuals are considered high risk, routine comprehensive assessments followed by tailored interventions are recommended [5]. Vitamin D supplementation should be limited to individuals with deficiencies, while prevention strategies may need to be tailored for those with specific medical conditions or those in resource-limited settings [5].

In India, SCI is not merely a medical condition but a life-altering social crisis. A single fall-related injury can lead to loss of livelihood, caregiver strain, and financial hardship for entire families. This underscores the need to view fall prevention not only as a medical priority but also as a public health and social justice imperative.

This year, the International Spinal Cord Society emphasizes fall prevention through education, safer environments, empowerment, and research [1]. The focus is on raising awareness, promoting safer design in daily settings, equipping people with SCI and their caregivers to manage risks, and encouraging innovation in rehabilitation and technology to protect spinal cord health [1]. On World SCI Day, India must seize the opportunity to convert awareness into action. Fall-prevention strategies should be embedded into all community outreach programs [5]. Physicians and physiatrists must lead efforts in screening for fall risk, counseling families, and initiating preventive interventions. Urban planning and construction should prioritize safety in housing, workplaces, and public spaces, while community campaigns must reinforce that falls are preventable, not inevitable.

Falls do not just break bones--they break lives. For India, where demographic changes are increasing the risk of fall-related SCI, prevention remains the most effective cure. World SCI Day serves as a reminder that every prevented fall preserves mobility, dignity, and independence, while also easing the socioeconomic burden on families and the healthcare system. The urgent task now is to move beyond awareness toward implementation, ensuring that fall prevention becomes a core part of India’s health and rehabilitation agenda.
